# Lupus Hepatitis: A Rare Manifestation Revealing Systemic Lupus Erythematosus

**DOI:** 10.7759/cureus.54003

**Published:** 2024-02-11

**Authors:** Hassnae Tkak, Anane Sara, Amal Hamami, Aziza Elouali, Abdeladim Babakhouya, Maria Rkain

**Affiliations:** 1 Department of Pediatrics, University Hospital Mohamed VI, Faculty of Medicine and Pharmacy, University Mohamed First, Oujda, MAR; 2 Department of Pediatrics, University Hospital Mohamed VI, Faculty of Medicine and Pharmacy, University Mohamed First,, Oujda, MAR; 3 Department of Pediatric Gastroenterology, University Hospital Mohamed VI, Faculty of Medicine and Pharmacy, University Mohamed First, Oujda, MAR

**Keywords:** corticosteroid, liver biopsy, auto-immune hepatitis, lupus hepatitis, systemic lupus erythematosus

## Abstract

Systemic lupus erythematosus (SLE) is a rare disease in children but is more severe than in adults. SLE may be associated with various non-specific hepatic manifestations, but subacute lupus hepatitis remains unusual and is rarely a mode of revelation. Diagnosis is based on a combination of clinical, laboratory, and histological findings after ruling out other causes of hepatitis, notably autoimmune hepatitis (AIH). We report the case of a young girl with undiagnosed SLE, which first revealed itself as liver involvement and progressed well on corticosteroid therapy. During the course of her illness, she presented with other manifestations that led us to think of SLE with lupus hepatitis.

## Introduction

Systemic lupus erythematosus (SLE) is a multisystem autoimmune disease of multifactorial origin secondary to genetic, immunological, and environmental factors [1]. Although rare in children, it is more severe than in adults and polymorphous in its presentation, with an unpredictable natural history and a higher frequency of renal manifestations [2]. The severity of pediatric lupus calls for early diagnosis and treatment to ensure optimal control of inflammation and avoid the associated morbidity and mortality, taking into account the child's development and growth. Liver involvement in patients with SLE is well documented but considered rare, with an incidence of 9.3%[3]. It may be associated with a number of non-specific hepatic manifestations, but sub-acute lupus hepatitis remains uncommon and rarely constitutes a revealing mode. Its diagnosis can be confirmed only after excluding viral, toxic, and immunological causes, and its treatment primarily involves the use of corticosteroids. The presentation of patients with SLE and AIH in terms of clinical and laboratory features is often similar, which complicates the diagnostic process.

## Case presentation

A 13-year-old girl, firstborn to a non-consanguineous couple, presented with cholestatic jaundice associated with pruritus evolving 10 days before admission in the context of apyrexia, anorexia, and weight loss. She had no significant medical history apart from a history of failure to thrive. On examination, she presented with mucocutaneous jaundice and delayed growth and weight. Her abdomen was distended, with a hepatomegaly 4 cm below the costal margin and a palpable spleen tip without lymphadenopathy. Additionally, the patient did not exhibit any joint involvement or rash. On laboratory examination, she had hepatic cytolysis, characterized by increased transaminases and cholestasis, marked by elevated levels of gamma-glutamyltransferase, alkaline phosphatase, Conjugated bilirubin, and total bilirubin, without hepatocellular failure (Table 1).

**Table 1:** Laboratory test results Compilation of biological results from liver function tests and complete blood count for our patient during the first and second hospitalizations before and after corticosteroid administration. SGOT: serum glutamic oxaloacelic transaminase; SGPT: serum glutamic pyruvic transaminase; GGT: Gamma-glutamyltransferase; ALP: alkaline phosphatase; TB: total bilirubin; CB: conjugated bilirubin

Laboratory parameter	The first hospitalization			The second hospitalization		Reference range
	At admission	15 days later	After one month of corticosteroid	At admission	After corticosteroid	
SGOT (UI/l)	410	895	28	290	25	5-34
SGPT (UI/l)	93	209	35	37	30	5-55
GGT (UI/l)	304	264	78	335	61	9-36
ALP (UI/L)	311	378	158	339	247	40-150
TB (mg/l)	156	266	9,9	55	2,1	2-12
CB (mg/l)	123	212	8	38	1	0-5
Haemoglobin (g/dl)	12,5	2,9	10,6	8,7	13,2	12-16
White blood cell (/µ)	4230	520	10230	3260	6160	4000-10000
Lymphocyte (/µl)	840	450	3850	1000	1160	2000-4000
Neutrophil (/µl)	3030	30	4980	2020	3990	1500-7000

Viral serologies were negative. Protein electrophoresis showed no abnormalities. The blood count revealed consistent lymphopenia. Abdominal ultrasound showed mild homogeneous hepatosplenomegaly. During her hospitalization, the child presented with fever and pallor, and in her laboratory findings, she had an inflammatory syndrome, with elevated C-reactive protein at 107 mg/l, hyperferritinemia at 2000 ug/l, hyperfibrinogenemia at 5,2 g/l, elevated sedimentation rate at 145 mm, and triglyceride at 2,7 g/l, a worsening liver work-up, and a non-regenerative cytopenia, neutropenia, profound hemolytic anemia with positive coombs test (Table 1), A myelogram found a deserted marrow with 2% blasts without hemophagocytosis.

**Figure 1:**
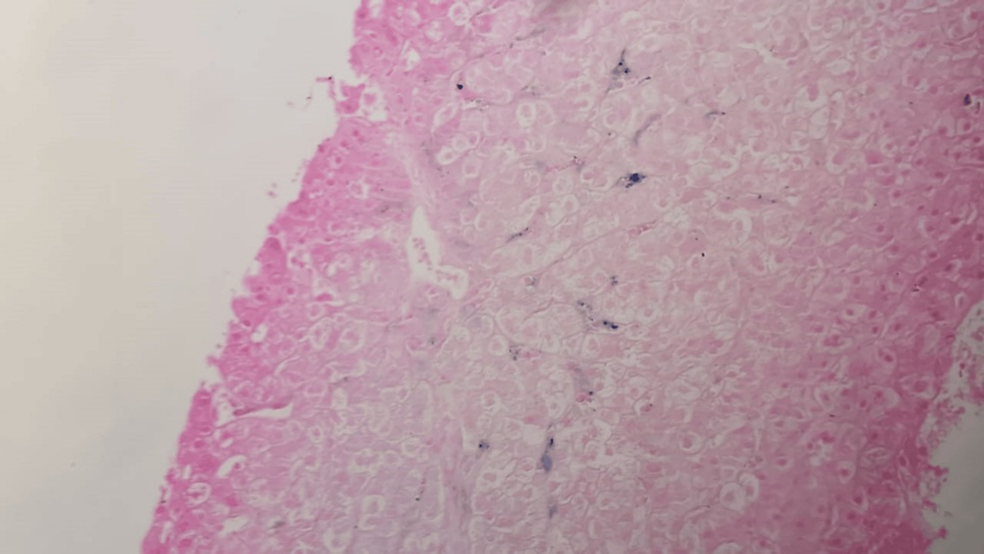
Histopathology image Histological examination of liver biopsy showing moderate hepatic cholestasis associated with minimal mesenchymal, without fibrosis or necrotic-inflammatory activity or signs of biliary disease or autoimmune hepatitis.

Osteo-medullary biopsy revealed hypoplastic marrow affecting both granulocytic and erythroblastic lineages with reactive megaloblastic hyperplasia secondary to a chronic inflammatory or liver disease. On the transthoracic ultrasound, there were no signs of myocarditis or pericardial effusion observed. As for the coronavirus disease 2019 (COVID-19) serologies, they were negative. Further investigation into autoimmune hepatitis revealed high levels of antinuclear antibodies (ANA) at 1280 UI/ml with a speckled pattern, anti-smooth muscle, anti-mitochondria, anti-LKM1, and anti-cytosol antibodies were negative. The total immunoglobulin (Ig)G level was slightly elevated at 13.7 g/l (Normal Value: 6,6-12,2 g/l). A liver biopsy showed no evidence of autoimmune hepatitis or chronic inflammation (Figure 1). Urinalysis revealed no proteinuria.

Given the severity of the clinical presentation, the patient was put on intravenous antibiotics, red blood cell concentrates, and a corticosteroid regimen. This corticosteroid protocol involved an initial bolus of methylprednisolone at a dosage of 30 mg/kg/day for three days, followed by an oral prednisone phase at a dose of 2 mg/kg/day for four weeks, gradually tapering over 12 weeks. Remarkably, both clinical and biological improvement was observed, with normalization of liver enzymes and cytopenias. The patient was subsequently lost to follow-up.

Sixteen months later, she was readmitted for a similar clinical presentation with an ascitic oedematous syndrome. Furthermore, she did not present neuropsychiatric symptoms, no skin or mucosal involvement, or alopecia. Biologically, she exhibited the same hepatic and hematological abnormalities as before: cholestasis and moderate cytolysis: associated with anemia, leukopenia, and lymphopenia (Table 1). Faced with the edema, a comprehensive biological evaluation was undertaken unveiling a pure nephrotic syndrome with nephrotic proteinuria, hypoalbuminemia, hypoproteinemia, and renal function was normal. An immunological work-up was performed revealing depleted complement levels with C3 at 0.36 g/l (Normal Value: 0,8-1,7 g/l] and C4 at 0.08 g/l (Normal Value: 0,13-0,46 g/l. The levels of ANA and anti-DNA antibodies were positive, whereas antiphospholipid antibodies, anti-SSA, anti-SSB, anti-SM, and anti-RNP antibodies were found to be negative. Confronted with this array of clinical and biological evidence, the diagnosis of lupus was considered. A renal biopsy was then performed, revealing a diffuse global proliferative lupus glomerulonephritis with active and chronic lesions classified as V+ IV-G, featuring an activity index of 7 and a chronicity index of 1. Meeting the criteria outlined by the EULAR/ACR-2019, the diagnosis of SLE was firmly established. The child received intravenous methylprednisolone at 30 mg/kg/day for 3 days, followed by oral prednisone at a dose of 1 mg/kg/day in combination with hydroxychloroquine 5 mg/kg/day, with ophthalmological monitoring. For stage IV lupus nephritis, the patient underwent a series of six monthly cycles of cyclophosphamide at a dosage of 500 mg/m2/cure alongside an angiotensin-converting enzyme inhibitor. The course was marked by both clinical and laboratory amelioration, illustrating a progressive resolution of hepatic, renal, and hematologic anomalies.

## Discussion

SLE is a multi-system autoimmune disease that generally affects adolescent girls, with a ratio of two to five females to one male before puberty and nine females to one male during the childbearing years [4]. It is characterized by the presence of circulating autoantibodies directed against autoantigens, with involvement of the kidneys, joints, central nervous system, skin, and rarely, liver [4]. Pediatric lupus is more severe than adult lupus in terms of renal complications, corticosteroid requirements, and consequences for development, growth, and quality of life [1].

In the literature, it is estimated that 75% of children with SLE will develop lupus nephropathy and nearly half of them will have renal involvement classified as stage IV by the World Health Organization (WHO) at the time of diagnosis [5,6]. The initial clinical manifestations of the disease vary widely, with a gradual, insidious onset. Non-specific symptoms are particularly misleading in adolescence. Arthritis, skin rash, and renal lesions are the most common conditions in children [7], while liver damage is considered rare. Although hepatic involvement is not considered a diagnostic criterion, disturbances in liver function tests are frequently observed, ranging from 23% to 60% according to various literature series [8,9]. The clinical and biological expression of this hepatic involvement can vary in severity, ranging from a simple disturbance in liver function, often asymptomatic, to a severe fulminant, life-threatening hepatitis [10]. These biological abnormalities are most often secondary to a drug-related, viral, metabolic, immunological cause, or thrombosis of the suprahepatic veins and are rarely related to specific lupus involvement, which is observed in 1 to 3% of SLE cases according to various authors [11,12]. Its diagnosis remains challenging as it requires the exclusion of the aforementioned diverse etiologies. Lupus hepatitis may occur concurrently with the diagnosis of lupus or later during a flare-up of the disease [13]. It may manifest as cytolysis with or without cholestasis and rarely as hepatic insufficiency [14].

In a retrospective monocentric study involving 73 patients with SLE, the prevalence of lupus hepatitis was 16.4%, with nearly half presenting with symptoms such as jaundice, hepatomegaly, abdominal pain, portal hypertension, and hepatic insufficiency at the time of diagnosis [13]. However, when considering all hepatic disturbances in lupus patients, this frequency is higher. Runyon et al. [9] found in a study of 206 lupus patients that 60% had abnormal liver test results, while Gibson and Myers [8] in a retrospective study of 81 lupus patients, reported a disturbance of the liver balance in 55% of cases. Biochemically, the most common abnormalities include elevated transaminases, with SGPT levels often exceeding SGOT levels, sometimes accompanied by increased alkaline phosphatase [13]. The frequency of anti-HCV antibodies in SLE varies between 2% and 20% in different studies [15,16], with a notable prevalence of cryoglobulinemia and severe liver involvement. Secondary antiphospholipid syndrome in SLE can lead to hepatic complications due to venous thrombosis in Budd-Chiari syndrome, although the diagnosis is not always straightforward [12,17].

Lupus hepatitis and autoimmune hepatitis (AIH) are two immunological conditions affecting the liver, sharing similar clinical, biological, and systemic manifestations, which complicates the diagnostic process [18,19]. Hepatomegaly is a significant and distinctive feature of AIH [20]. Although histopathological characteristics are useful for diagnosing AIH, they do not rule out lupus hepatitis. Physicians must be aware of both liver conditions, as early diagnosis and corticosteroid treatment are crucial for preventing mortality. The development of lupus hepatitis is attributed to the expression of pro-inflammatory cytokines, regulated by anti-ribosomal P antibodies, which can serve as a serological diagnostic marker to differentiate lupus hepatitis. Studies in the literature have identified a correlation between liver involvement and the presence of these antibodies in 10 to 40% of SLE patients, demonstrating an overall sensitivity and specificity of 23.1% and 99%, respectively [21]. This clinical-biological correlation was also described for anti-Sm, anti-tRNA, anti-thyroglobulin, and anti-microsomal antibodies [22,23], in contrast to ANAs, which characterize both conditions [21]. Additional biological markers for diagnosing lupus hepatitis include low levels of complement 3, anti-double-stranded DNA, and non-specific inflammation with histologically identified fatty degeneration. In resource-limited settings where liver biopsy is not feasible, serological tests such as positive ANA and anti-ribosomal P antibodies can serve as valuable diagnostic markers for lupus hepatitis [20].

## Conclusions

Lupus hepatitis is a rare manifestation of systemic lupus erythematosus that should not be overlooked and seldom serves as a revealing mode. Diagnosis relies on a comprehensive evaluation of clinical, biological, and histological evidence after ruling out other causes of hepatitis. The prognosis is typically favorable with systemic corticosteroid therapy, rarely requiring the use of immunosuppressants. All published studies concur on its favorable outcome.
